# Metabolic Regulation of Macrophages by SIRT1 Determines Activation During Cholestatic Liver Disease in Mice

**DOI:** 10.1016/j.jcmgh.2021.12.010

**Published:** 2021-12-22

**Authors:** Anna Isaacs-Ten, Mar Moreno-Gonzalez, Caitlin Bone, Andre Martens, Federico Bernuzzi, Tobias Ludwig, Charlotte Hellmich, Karsten Hiller, Stuart A. Rushworth, Naiara Beraza

**Affiliations:** 1 Gut Microbes and Health Institute Strategic Programme, Quadram Institute Bioscience, Norwich, United Kingdom; 2 Department of Bioinfomatics and Biochemistry, Braunschweig Integrated Center of Systems Biology, Braunschweig, Germany; 3 Food Innovation and Health Institute Strategic Programme, Quadram Institute Bioscience, Norwich, United Kingdom; 4 Norwich Medical School, University of East Anglia, Norwich Research Park, Norwich, United Kingdom; 5 Department of Haematology, Norfolk and Norwich University Hospitals NHS Trust, Norwich, United Kingdom; 6 Computational Biology of Infection Research, Helmholtz Centre for Infection Research, Braunschweig, Germany

**Keywords:** SIRT1, Metabolism, Macrophages, Inflammasome, Cholestasis, ALT, alanine aminotransferase, AST, aspartate aminotransferase, BDL, bile duct ligation, BSA, bovine serum albumin, BMDM, bone marrow–derived macrophage, IF, immunofluorescence, IL, interleukin, LPS, lipopolysaccharide, mTOR, mammalian target of rapamycin, NF-κB, nuclear factor kappa B, PBS, phosphate-buffered saline, PRR, pathogen recognition receptor, S6K1, S6 kinase 1, SIRT1, sirtuin 1, SIRT^oe^, sirtuin 1 overexpressing, TCA, tricarboxylic acid, WT, wild-type

## Abstract

**Background & Aims:**

Inflammation is the hallmark of chronic liver disease. Metabolism is a key determinant to regulate the activation of immune cells. Here, we define the role of sirtuin 1 (SIRT1), a main metabolic regulator, in controlling the activation of macrophages during cholestatic liver disease and in response to endotoxin.

**Methods:**

We have used mice overexpressing SIRT1, which we treated with intraperitoneal lipopolysaccharides or induced cholestasis by bile duct ligation. Bone marrow–derived macrophages were used for mechanistic in vitro studies. Finally, PEPC-Boy mice were used for adoptive transfer experiments to elucidate the impact of SIRT1-overexpressing macrophages in contributing to cholestatic liver disease.

**Results:**

We found that SIRT1 overexpression promotes increased liver inflammation and liver injury after lipopolysaccharide/GalN and bile duct ligation; this was associated with an increased activation of the inflammasome in macrophages. Mechanistically, SIRT1 overexpression associated with the activation of the mammalian target of rapamycin (mTOR) pathway that led to increased activation of macrophages, which showed metabolic rewiring with increased glycolysis and broken tricarboxylic acid cycle in response to endotoxin in vitro. Activation of the SIRT1/mTOR axis in macrophages associated with the activation of the inflammasome and the attenuation of autophagy. Ultimately, in an in vivo model of cholestatic disease, the transplantation of SIRT1-overexpressing myeloid cells contributed to liver injury and fibrosis.

**Conclusions:**

Our study provides novel mechanistic insights into the regulation of macrophages during cholestatic disease and the response to endotoxin, in which the SIRT1/mTOR crosstalk regulates macrophage activation controlling the inflammasome, autophagy and metabolic rewiring.

## Graphical abstract

**Figure:**
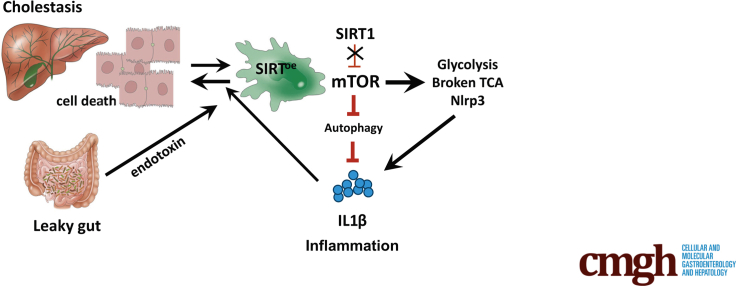

**Summary**

Here, we describe that the SIRT1/mTOR axis regulates metabolic rewiring, inflammasome activation, and autophagy in macrophages, in which SIRT1 overexpression actively contributes to aggravate cholestatic liver disease progression in mice.

During chronic liver disease, the accumulation of dying cells in the liver together with the translocation of bacteria (products) from a leaky gut contribute to disease progression by sustaining inflammation.^1^, ^2^, ^3^ Macrophages are the first line of defense to respond to bacteria and to remove cellular debris and thus play an essential role during chronic disease, in which proinflammatory macrophages infiltrate the liver and contribute to disease progression and fibrosis.^4^, ^5^, ^6^ The mechanisms controlling macrophage function are complex and it is now apparent that metabolic rewiring, the regulation of the inflammasome and autophagy are essential to regulate macrophage activation.^7^, ^8^, ^9^, ^10^, ^11^

In the last decade, the development of new metabolomic techniques has contributed to establishing the role of metabolism in regulating macrophage function showing increased glycolysis and a rewired tricarboxylic acid (TCA) cycle during activation.^7^

The influence of metabolic reprogramming in controlling macrophage activation expands to the regulation of the inflammasome, as glycolysis regulates Nlrp3-dependent inflammasome activation^8^ while succinate accumulating from a broken TCA promotes interleukin (IL)-1β expression.^9^ The inflammasome, a multiprotein complex assembled in the cytosol after pathogen recognition receptors (PRRs) engage with bacteria (products)^12^ is activated during human^2^ and murine cholestatic disease, in which it mediates the progression of the disease as we and others have described.^1^^,^^2^^,^^13^, ^14^, ^15^

Recent evidence supports the role of key metabolic regulators, including the mammalian target of rapamycin (mTOR), in modulating inflammation.^16^^,^^17^ mTOR is a protein kinase formed by 2 subunits with differential functions: mTORC1 and mTORC2.^18^^,^^19^ mTORC1 regulates the activation of the Nlrp3 inflamamsome in macrophages by promoting glycolysis.^8^ Additionally, mTORC1 inhibits autophagy^11^ and thus controls the activation of the inflammasome.^20^

Sirtuin 1 (SIRT1) is a multifaceted histone deacetylase that controls cell energy and metabolism.^21^ SIRT1 was initially described to mediate the benefits of calorie restriction in prolonging the lifespan of lower organisms.^22^ Those findings were further challenged and proved to be tissue-specific,^23^ underlining the complexity of the role of SIRT1 in controlling mammalian cell function. We and others have demonstrated that SIRT1 is highly expressed in human liver tumors.^24^, ^25^, ^26^ More recently, we described that SIRT1 is upregulated in the liver of patients with chronic cholestatic disease and in cholestatic mice, in which we showed that the overexpression of SIRT1 contributed to liver injury and fibrosis.^27^ In that context, we found increased inflammation in SIRT1-overexpressing (SIRT^oe^) mice, which contrasts with its previously described anti-inflammatory effects.^28^, ^29^, ^30^, ^31^, ^32^

Here, we further define the role of SIRT1 in regulating macrophage activation during cholestasis and in response to endotoxin. Our results provide mechanistic evidence of the role of SIRT1 in regulating macrophage activation by modulating cell metabolism, the inflammasome, and autophagy. Ultimately, we demonstrate that the overexpression of SIRT1 in macrophages contributes to the aggravation of cholestasis-mediated liver injury by promoting inflammation and fibrosis.

## Results

### Overexpression of SIRT1 Promotes Increased Inflammasome Activation in the Liver During Cholestasis

We previously described that SIRT1 expression was increased in the livers from primary sclerosing cholangitis and primary biliary cholangitis patients as well as in mice following bile duct ligation (BDL) and that the overexpression of SIRT1 contributed to cholestatic disease progression in SIRT^oe^ mice after BDL.^27^

**Figure 1:**
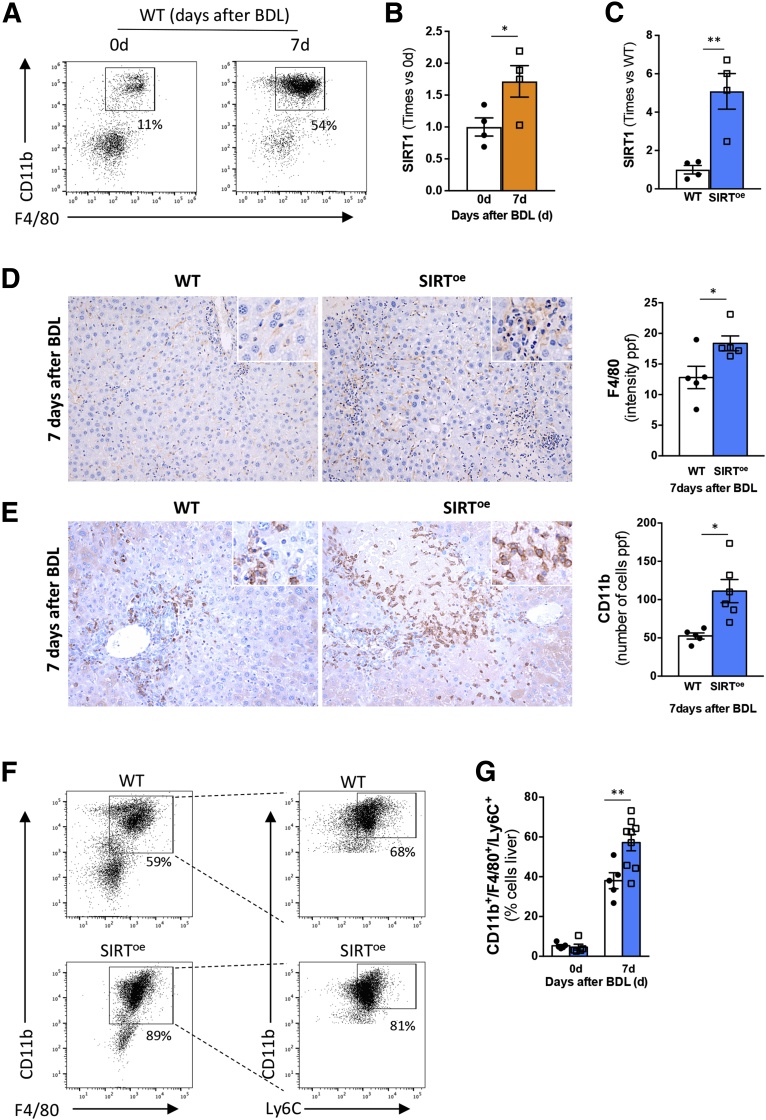
**The overexpression of SIRT1 promotes inflammation in mice after BDL.** (*A*) Cell sorting strategy to isolate CD11b^+^/F4/80^+^ macrophages from livers from WT mice before and 7 days after BDL. (*B*) Quantitative polymerase chain reaction analysis on isolated macrophages from livers showing increased SIRT1 expression after BDL in WT mice. (*C*) Quantitative polymerase chain reaction analysis showing increased SIRT1 expression in macrophages isolated from SIRT^oe^ mice compared with WT mice. (*D*) Immunohistochemistry using an anti-F4/80 and (*E*) anti-CD11b antibody in paraffin-embedded liver sections and further quantification showing increased presence of macrophages in SIRT^oe^ mice compared with WT mice after BDL. (*F*) Gating strategy to detect increased presence of infiltrating macrophages in livers from SIRT^oe^ mice compared with WT mice 7 days after BDL by fluorescence-activated cell sorting. (*G*) Quantification after fluorescence-activated cell sorting analysis of immune cells isolated from livers showing increased infiltration of macrophages 7 days after BDL. n = 4–9 animals/treatment group were analyzed. Values are mean ± SEM. ∗*P <* .05, ∗∗*P <* .01 (WT vs SIRT^oe^). (*D*, *E*) Representative microscopical images are shown at ×10.

Here, we show that, in addition to the up regulation in hepatocytes we previously described,^27^ SIRT1 expression is also increased in macrophages (CD11b^+^/F4/80^+^) isolated from livers of wild-type (WT) mice at 7 days after BDL (Figure 1*A* and *B*).

Next, we found that SIRT^oe^ mice (Figure 1*C*) had increased inflammation, characterized by the higher presence of F4/80-positive (Figure 1*D*) and CD11b-positive (Figure 1*E*) cells in the liver compared with WT animals at 7 days after BDL. Fluorescence-activated cell sorting analysis confirmed the increased infiltration of macrophages (CD11b^+^/F4/80^+^/Ly6C^+^) in livers from SIRT^oe^ mice compared with WT mice at 7 days after BDL (Figure 1*F* and *G*).

**Figure 2:**
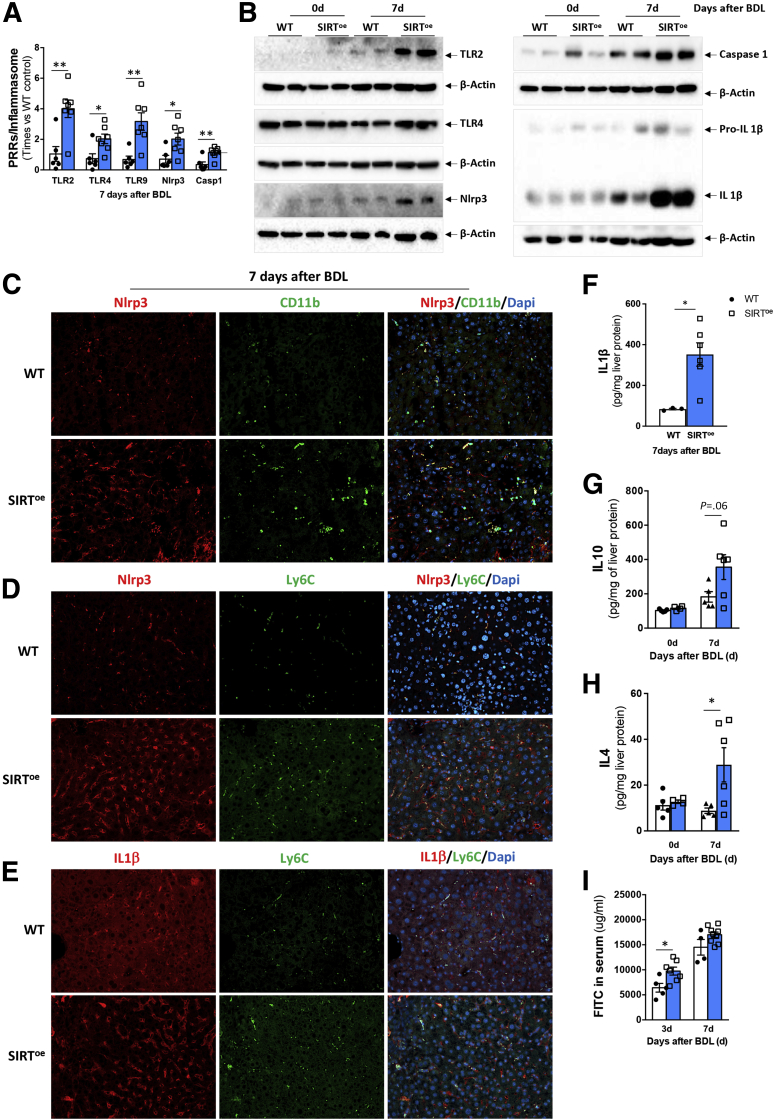
**The overexpression of SIRT1 associates with increased Nlrp3 inflammasome activation in CD11b**^**+**^**cells and Ly6C**^**+**^**macrophages in the liver in response to BDL.** (*A*) Quantitative polymerase chain reaction analysis of liver extracts supporting increased expression of pathogen receptors (TLR) and inflammasome components. (*B*) Western blot analysis confirming increased TLRs, Nlrp3, cleaved caspase-1, and IL-1β, supporting activation of the inflammasome in SIRT^oe^ livers after BDL. Immunofluorescence showing colocalization (*C*) of Nlrp3 (red) and CD11b (green), (*D*) of Nlrp3 (red) and Ly6C (green), and (*E*) of IL-1β (red) and Ly6C (green) in liver sections 7 days after BDL. Cell nuclei are in blue stained with DAPI. (*F*) Quantitative polymerase chain reaction analysis showing increased IL-1β expression in macrophages isolated from SIRT^oe^ mice compared with WT mice. (*G*) Enzyme-linked immunosorbent assay on whole liver protein extracts showing increased expression of IL-10 and (*H*) IL-4 in SIRT^oe^ livers compared with WT mice at 7 days after BDL and control mice. (*I*) Quantification of circulating FITC in serum samples from WT and SIRT^oe^ mice 3 and 7 days after BDL supporting increased intestinal permeability in SIRT^oe^ animals. n = 4–9 animals/treatment group were analyzed. Values are mean ± SEM. ∗*P <* .05, ∗∗*P <* .01 (WT vs SIRT^oe^). (*C–E*) Representative microscopical images are shown at ×20 magnification.

Macrophages sense dying cells and bacteria via PRRs (eg, TLR) that activate the Nlrp3 inflammasome to promote the proteolytic cleavage of pro-caspase-1 into caspase-1 that further cleaves pro-ILβ into IL-1β, its mature form.^12^ During cholestasis, the activation of the Nlrp3 inflammasome in macrophages plays a key role in contributing to disease progression.^2^^,^^13^^,^^14^^,^^33^ In line with the more severe phenotype we observed in SIRT^oe^ mice after BDL, we found increased gene expression of TLR-2, TLR-4 and TLR-9, Nlrp3, and caspase-1 genes in SIRT^oe^ mice compared with WT mice 7 days after BDL (Figure 2*A*). These results were confirmed by Western blot analysis showing an apparent increase in TLR2, and Nlrp3, as well as higher protein expression of cleaved caspase-1 and cleaved IL-1β in livers from SIRT^oe^ mice after BDL compared with WT mice, while mild regulation of TLR4 was observed (Figure 2*B*).

Previous studies have shown that the activation of the inflammasome during cholestasis is restricted to nonparenchymal cells and specifically to macrophages in primary sclerosing cholangitis patients and in mice after BDL.^2^^,^^13^^,^^14^^,^^33^ Our results obtained from immunofluorescence (IF) staining of liver sections confirmed that Nlrp3 colocalized with CD11b-positive (Figure 2*C*) and Ly6C-positive (Figure 2*D*) cells in livers from WT and SIRT^oe^ mice 7 days after BDL. In addition, IF analyses confirmed the colocalization of IL-1β  in Ly6C positive cells in livers at 7 days after BDL (Figure 2*E*). Ultimately, we isolated CD11b^+^/F4/80^+^ macrophages from mouse livers at 7 days after BDL and found that SIRT^oe^-isolated cells had increased IL-1β  protein expression compared with WT mice (Figure 2*F*). Overall, these results support that the inflammasome activation we observed in total liver samples was originated mainly in macrophages as previously described.^2^^,^^13^^,^^14^^,^^33^

Interestingly, the proinflammatory phenotype observed was accompanied by an increase in IL-10 and IL-4 expression in livers from SIRT^oe^ mice after BDL compared with WT mice (Figure 2*G* and *H*), supporting that a pro- and anti-inflammatory phenotype can coexist in macrophages beyond the classical M1/M2 polarization in the liver during disease.^34^, ^35^, ^36^

Chronic liver disease, including cholestasis, associates with the disruption of the intestinal barrier function characterized by increased intestinal permeability (leaky gut).^1^^,^^3^^,^^37^^,^^38^ This allows the translocation of bacteria (and their products [ie, endotoxins]) into the liver via the systemic circulation,^39^ aggravating inflammation and thus disease progression.^1^ The increased inflammation and activation of the inflammasome we observed in SIRT^oe^ mice could associate with the higher presence of bacteria (products) in the liver due to increased intestinal permeability.^1^^,^^38^ In accordance, we detected higher FITC-labeled dextran in circulation in SIRT^oe^ mice at 3 days after BDL compared with WT mice denoting increased intestinal leakage that could contribute to the increased liver inflammation observed (Figure 2*I*).

Overall, we here describe that during cholestasis SIRT1 is upregulated and its overexpression associates with increased liver inflammation and inflammasome activation in macrophages after BDL.

### Endotoxin Promotes Increased Liver Injury and Inflammation in the Liver of SIRT^oe^ Mice

Following the leaky gut hypothesis,^1^^,^^3^^,^^37^^,^^38^ the higher inflammation/inflammasome activity found in SIRT^oe^ mice after BDL could result from the increased liver injury, the higher translocation of bacterial products from a more permeable gut, or the intrinsic overactivation of macrophages.

**Figure 3:**
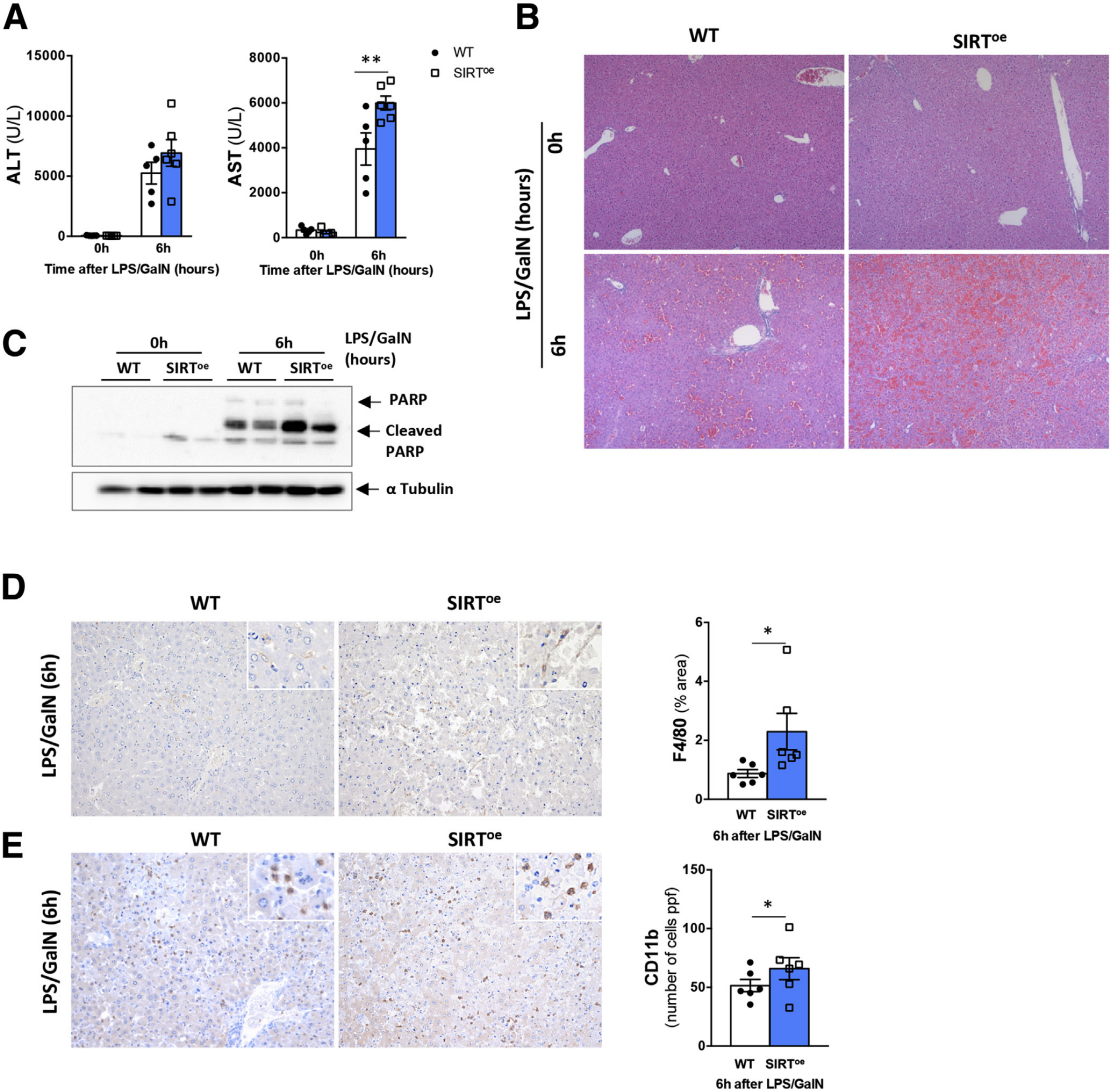
**The overexpression of SIRT1 promotes increased liver injury and inflammasome activation in response to endotoxin.** (*A*) Quantification of liver injury blood markers (ALT and AST) indicates increased liver injury in SIRT^oe^ mice 6 hours after LPS/GalN treatment. (*B*) Hematoxylin and eosin staining of liver sections from WT and SIRT^oe^ mice confirms more severe parenchymal damage in LPS/GalN-SIRT^oe^ mice. (*C*) Western blot analysis supported increased apoptosis in LPS/GalN-SIRT^oe^ mice compared with LPS/GalN-WT mice. Immunohistochemistry in paraffin-embedded liver sections using an (*D*) anti-F4/80 and (*E*) anti-CD11b antibody, followed by quantification show increased presence of macrophages in livers from SIRT^oe^ mice compared with WT mice 6 hours after LPS/GalN. n = 5–6 animals/treatment group were analyzed. Values are mean ± SEM. ∗*P <* .05, ∗∗*P <* .01 (WT vs SIRT^oe^). Representative microscopical images are shown at (*B*) ×4 and (*D*, *E*) ×10.

To determine this, we treated WT and SIRT^oe^ mice with lipopolysaccharide (LPS)/GalN. As shown in Figure 3*A*, SIRT^oe^ mice showed signs of increased liver injury as evidenced by higher alanine aminotransferase (ALT) and aspartate aminotransferase (AST) levels 6 hours after LPS/GalN. Histopathological analysis on liver sections confirmed the more profound parenchymal damage in SIRT^oe^ mice 6 hours after LPS/GalN compared with WT animals (Figure 3*B*). Further analysis of PARP protein cleavage supported the increased liver apoptosis in SIRT^oe^ mice after LPS/GalN (Figure 3*C*).

Next, we determined the impact of LPS/GalN treatment on the inflammatory response in the liver. Our results show that SIRT^oe^ mice have increased presence of F4/80-positive (Figure 3*D*) and CD11b-positive (Figure 3*E*) cells in the liver at 6 hours after LPS/GalN treatment compared with WT mice.

**Figure 4:**
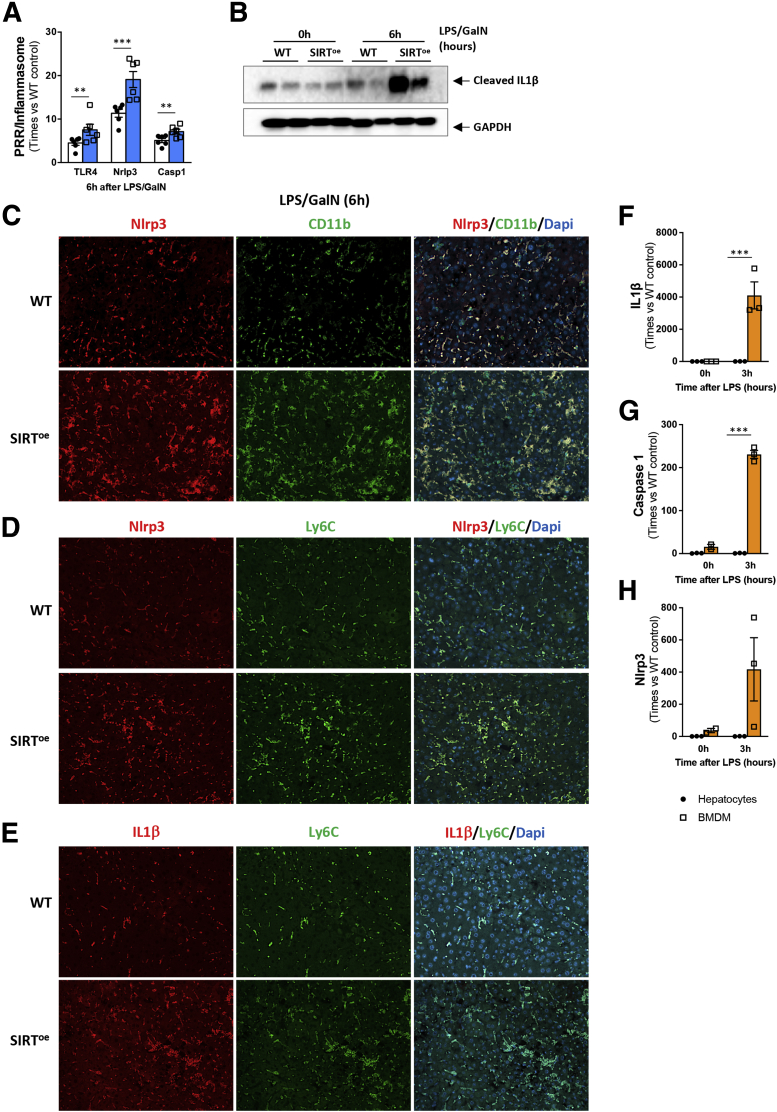
**The overexpression of SIRT1 promotes inflammasome activation in response to endotoxin.** (*A*) Quantitative polymerase chain reaction analysis of liver extracts indicates increased expression of TLR and inflammasome components. (*B*) Western blot analysis showing higher expression of cleaved IL-1β supporting activation of the inflammasome in SIRT^oe^ livers 6 hours after LPS/GalN. Immunofluorescence showing colocalization (*C*) of Nlrp3 (red) and CD11b (green), (*D*) of Nlrp3 (red) and Ly6C (green), and (*E*) of IL-1β (red) and Ly6C (green) in liver sections from WT and SIRT^oe^ mice 6 hours after LPS/GalN. Primary hepatocytes and BMDMs from WT mice were exposed to 100 ng/mL of LPS and (*F*) IL-1β , (*G*) caspase-1, and (*H*) Nlrp3 gene expression was analyzed by quantitative polymerase chain reaction showing increased response in BMDMs compared with hepatocytes, which show only a marginal response. Slides were mounted on a solution containing DAPI (blue) staining cell nuclei. n = 5–6 animals/treatment group were analyzed. Values are mean ± SEM. ∗∗*P <* .01, ∗∗∗*P <* .001 (WT vs SIRT^oe^). (*C–E*) Representative microscopical images are shown at ×20 magnification. Results from in vitro experiments are representative analysis of n = 3 replicates per timepoint, per cell type. Values are mean ± SEM. ∗*P <* .05, ∗∗*P <* .01 (WT vs SIRT^oe^).

In line with this, LPS/GalN treatment resulted in increased TLR4, Nlrp3, and caspase-1 gene expression in SIRT^oe^ mice compared with WT animals (Figure 4*A*). Our results showing higher cleaved IL-1β protein (Figure 4*B*) support the stronger activation of the inflammasome in livers from mice overexpressing SIRT1 after LPS/GalN compared with WT mice. IF costainings of Nlrp3 and CD11b (Figure 4*C*) as well as costainings of Ly6C and Nlrp3 (Figure 4*D*) and Ly6C and IL-1β  (Figure 4*E*) confirmed the localization of the inflammasome in macrophages in mouse livers after LPS/GalN treatment, which was higher in SIRT^oe^ mice compared with WT mice.

Further in vitro analyses comparing the expression levels of inflammasome components in hepatocytes and macrophages showed marginal response to LPS in hepatocytes compared with BMDMs (Figure 4*F–H*). This supports macrophages as the main source of inflammasome-activation we observed in response to LPS in vivo.

**Figure 5:**
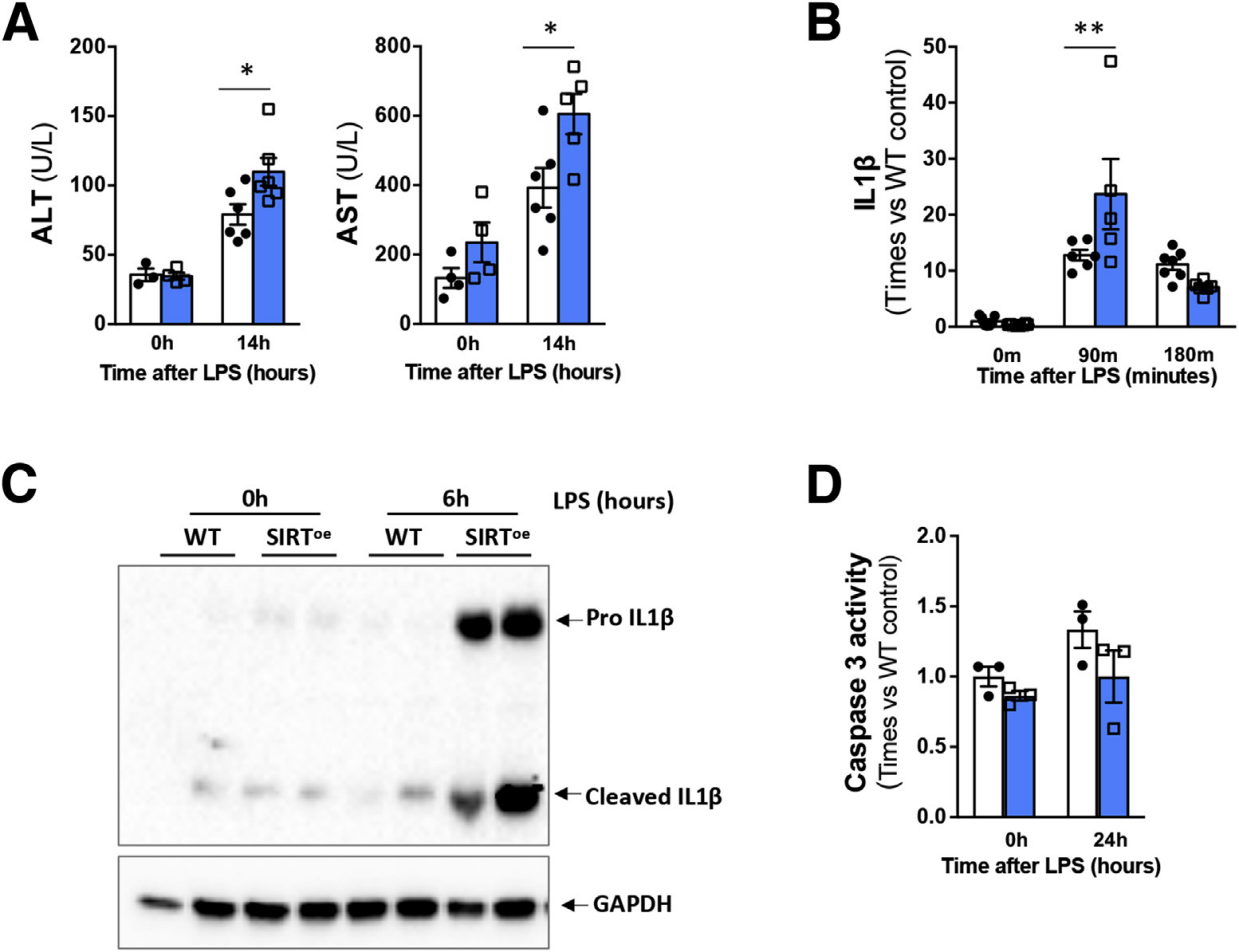
**The overexpression of SIRT1 promotes increased liver injury and inflammasome activation in response to LPS.** (*A*) Quantification of liver injury markers (ALT and AST) in serum samples indicates increased liver injury in SIRT^oe^ mice 14 hours after LPS treatment. (*B*) Quantitative polymerase chain reaction analysis and (*C*) Western blot analysis shows increased IL-1β  gene expression and cleavage, overall supporting the activation of the inflammasome in SIRT^oe^ livers 6 hours after LPS. (*D*) Caspase-3 activity was quantified showing mild impact of LPS in WT hepatocyte cell death that was not increased in SIRT^oe^ primary hepatocytes. n = 4–7 animals/treatment group were analyzed. Values are mean ± SEM. ∗*P <* .05, ∗∗*P <* .01 (WT vs SIRT^oe^). Results from in vitro experiments are representative analysis of n = 3 replicates per time point.

The increased susceptibility to LPS-induced liver injury and inflammation in SIRT^oe^ mice was confirmed in mice treated with LPS alone for up to 14 hours, in which we found increased ALT/AST levels (Figure 5*A*), elevated IL-1β gene expression (Figure 5*B*), and protein cleavage (Figure 5*C*) when compared with WT mice.

Interestingly, the analysis of isolated primary hepatocytes exposed to endotoxin in vitro, showed that LPS had a comparable mild impact on hepatocyte cell death in SIRT^oe^ and WT cells (Figure 5*D*). This result suggests that the exacerbated liver injury observed in SIRT^oe^ mice in vivo may result from the increased inflammatory response mediated by activated macrophages, rather than from a direct effect of LPS on hepatocyte cell death.

### SIRT1 Overexpression Concurs With mTORC1 Activation and Contributes to the Activation of Macrophages by Activating the Inflammasome and Attenuating Autophagy

**Figure 6:**
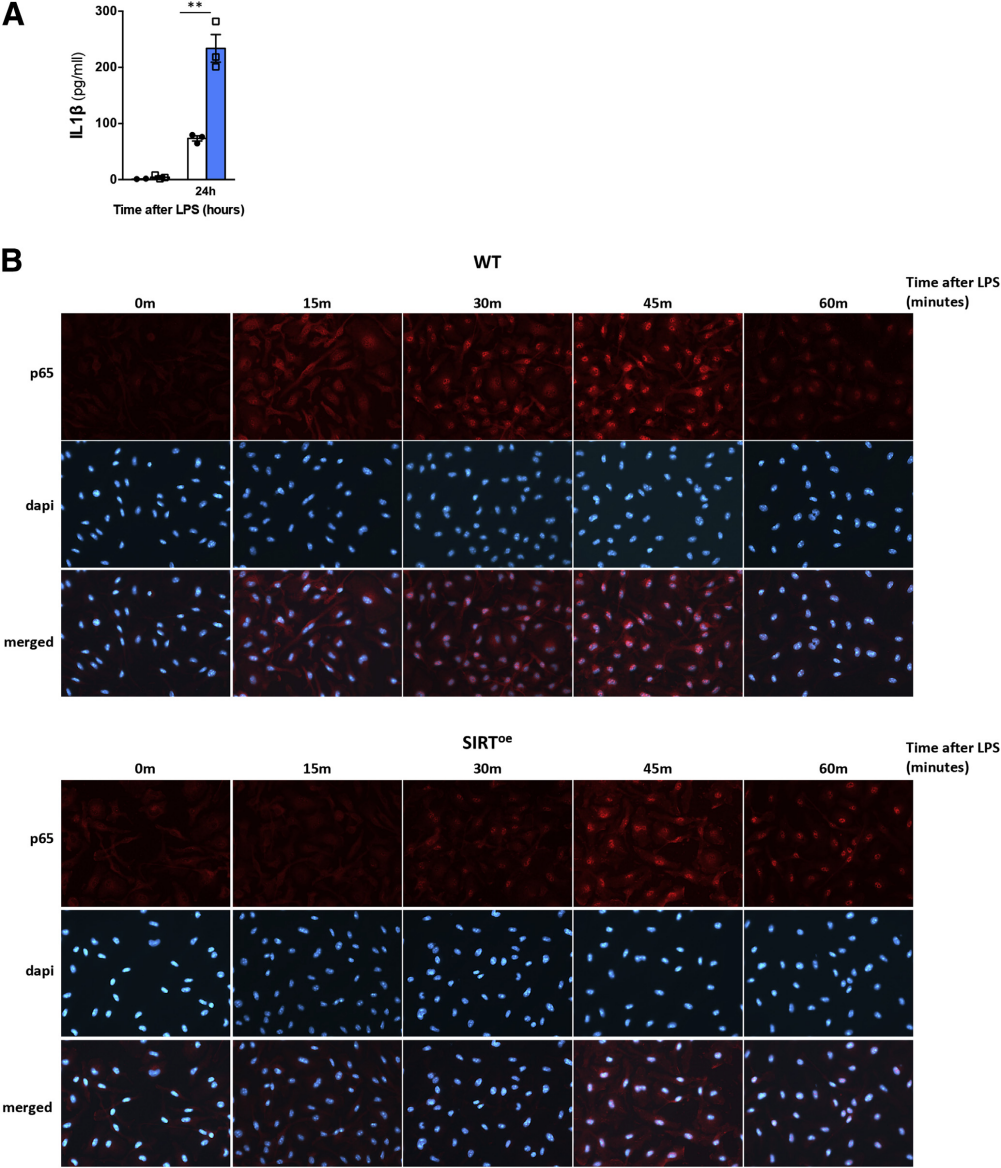
**The overexpression of SIRT1 promotes proinflammatory phenotype in BMDMs associated with activation of mTOR signaling and decreased autophagy.** (*A*) Increased IL-1β secretion detected by enzyme-linked immunosorbent assay on supernatants of BMDMs treated with 100 ng/mL LPS for 24 hours. (*B*) Immunocytochemistry on cultured BMDMs using an anti-p65 antibody (red) indicates attenuated and delayed translocation of p65 into the nucleus in SIRT^oe^ BMDMs after LPS. Experiments were done twice in triplicate. Values are mean ± SEM. ∗*P <* .05, ∗∗*P <* .01, ∗∗∗*P <* .001 (WT vs SIRT^oe^). Representative microscopical images are shown at ×20 magnification.

To confirm the role of SIRT1 in controlling macrophage activation and the underlying mechanisms mediating this effect we isolated and differentiated bone marrow–derived macrophages (BMDMs) from WT and SIRT^oe^ mice. Stimulation with LPS increased IL-1β expression in SIRT^oe^ BMDMs compared with WT cells (Figure 6*A*), which challenges the previously described anti-inflammatory role of SIRT1 based on the attenuation of nuclear factor kappa B (NF-κB) activity.^31^ To determine this, we performed immunocytochemistry analysis on LPS-BMDMs that evidenced a delayed nuclear translocation of p65 in SIRT^oe^ BMDMs after LPS compared with WT cells rather than a complete inhibition (Figure 6*B*).

**Figure 7:**
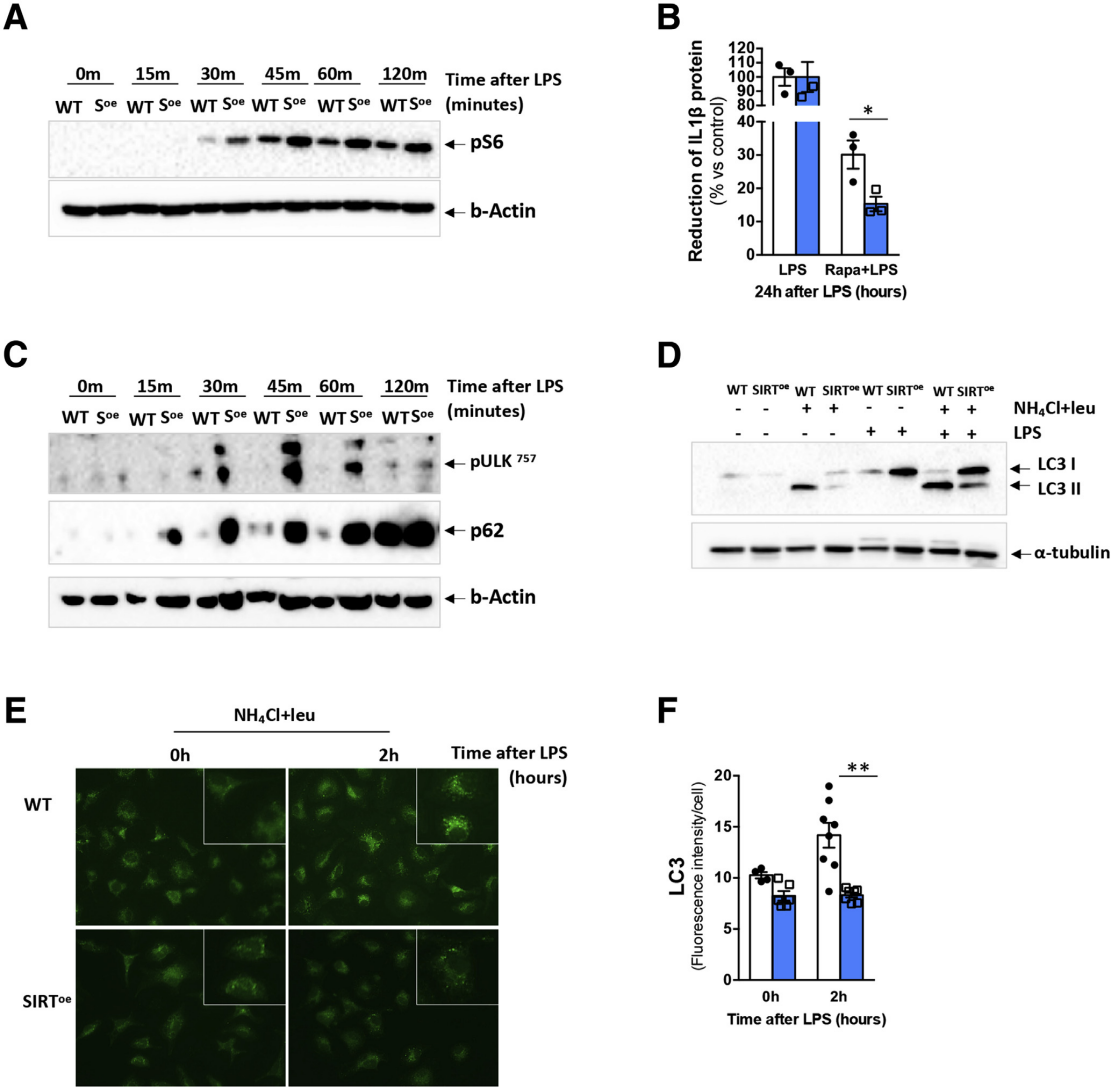
**The overexpression of SIRT1 promotes activation of mTOR signaling and decreased autophagy in BMDMs.** (*A*) Western blot analysis shows earlier and increased phosphorylation of S6 in SIRT^oe^ BMDMs after LPS. (*B*) Enzyme-linked immunosorbent assay of BMDM supernatants that were pretreated with 50 nM of rapamycin 1 hour before LPS treatment for 24 hours showed stronger decrease in IL-1β secretion in SIRT^oe^ BMDMs compared with WT cells (expressed in % of reduction). (*C*) Western blot analysis showing increased phosphorylation of ULK in serine 757 and increased accumulation of p62 in SIRT^oe^ BMDMs in response to LPS compared with WT cells. To evaluate autophagy, lysosomal proteolysis was inhibited by pretreating cells with 20 mM NH_4_Cl and 100 μM leupeptin at 2 hours before LPS treatment. In these conditions, (*D*) Western blot analysis shows increased accumulation of lipidated LC3II in LPS/WT BMDMs compared with LPS/SIRT^oe^ cells that shows lower LC3II but higher LC3I expression. (*E*) Attenuated autophagy in LPS/ SIRT^oe^ BMDMs was confirmed by ICC using anti-LC3 antibody (green). (*F*) Further quantification shows decreased presence of fluorescent-labeled LC3 compared with the WT mice, which show increased LC3 staining 2 and 3 hours after LPS. Experiments were done twice in triplicate. Values are mean ± SEM. ∗*P <* .05, ∗∗*P <* .01 (WT vs SIRT^oe^). Representative microscopical images are shown at ×20 magnification.

The activation of the inflammasome can be regulated by different signaling pathways, including mTORC1.^8^ mTORC1 activation leads to the downstream activation of S6 kinase 1 (S6K1) that phosphorylates the ribosomal S6 protein.^18^^,^^19^ Our results showed that pS6 was phosphorylated earlier in SIRT^oe^ BMDMs than in WT cells after LPS treatment, indicating increased mTORC1 activation in SIRT^oe^ cells (Figure 7*A*). Furthermore, the inhibition of mTORC1 with rapamycin showed a more profound attenuation of IL-1β production in SIRT^oe^ cells compared with WT cells after LPS, supporting the higher dependency of SIRT^oe^ cells on the mTOR pathway to secrete IL-1β in response to LPS compared with WT BMDMs (Figure 7*B*).

The proinflammatory function of mTORC1 is also supported by its capacity to inhibit autophagy via the phosphorylation of ULK in serine 757.^11^ In turn, autophagy negatively regulates inflammation via inhibiting the inflammasome activation.^40^ Accordingly, we found increased presence of pULK^757^ in SIRT^oe^ BMDMs than in WT cells after LPS treatment, confirming increased activation of the mTOR pathway in SIRT^oe^ BMDMs (Figure 7*C*). p62/SQSTM1 (herein p62) is an adaptor protein that contributes to the mTORC1-mediated regulation of autophagy. Additionally, p62 is a substrate of autophagy that accumulates when autophagy is impaired.^41^ Our results show strong accumulation of p62 in SIRT^oe^ BMDMs compared with WT cells after LPS treatment, pointing to the attenuation of autophagy in the SIRT^oe^ cells (Figure 7*C*).

During autophagy, cytosolic material is engulfed in the autophagosome, a double-membrane structure coated with lipidated LC3II that directs it to the lysosome for fusion into an autolysosome.^11^ Our results show that LPS induced accumulation of the lipidated LC3II subunit in WT BMDMs after inhibition of the autophagy flux with ammonium chloride/leupeptin pretreatment, while SIRT^oe^ cells had higher expression of LC3I and lower LC3II (Figure 7*D*). Immunocytochemistry analysis and further quantification confirmed the increased presence of LC3 puncta, consistent with phagosome formation, in WT BMDMs after LPS treatment (Figure 7*E* and *F*), supporting the attenuation of autophagy in SIRT^oe^ BMDMs.

Overall, our in vitro results indicate that the overexpression of SIRT1 associates with the concomitant activation of mTORC1 that leads to the activation of the inflammasome and the attenuation of autophagy, overall promoting the proinflammatory activity of macrophages.

### Overexpression of SIRT1 Promotes Metabolic Rewiring of TCA Cycle and Increased Glycolysis in Macrophages

Metabolic reprogramming is key to control the inflammatory response in macrophages. A hallmark of this reprogramming is the rewiring of the TCA cycle, the so-called broken TCA cycle, which enables the accumulation of metabolic intermediates citrate, succinate, and fumarate.^7^^,^^42^ It is now obvious that these metabolites regulate the inflammatory response (ie, IL-1β). Thus, citrate is key for the synthesis of itaconate, a hallmark of macrophage activation that contributes to accumulation of intracellular succinate,^43^, ^44^, ^45^ essential to sustain inflammation by promoting increased IL-1β levels via glycolysis in macrophages.^9^

**Figure 8:**
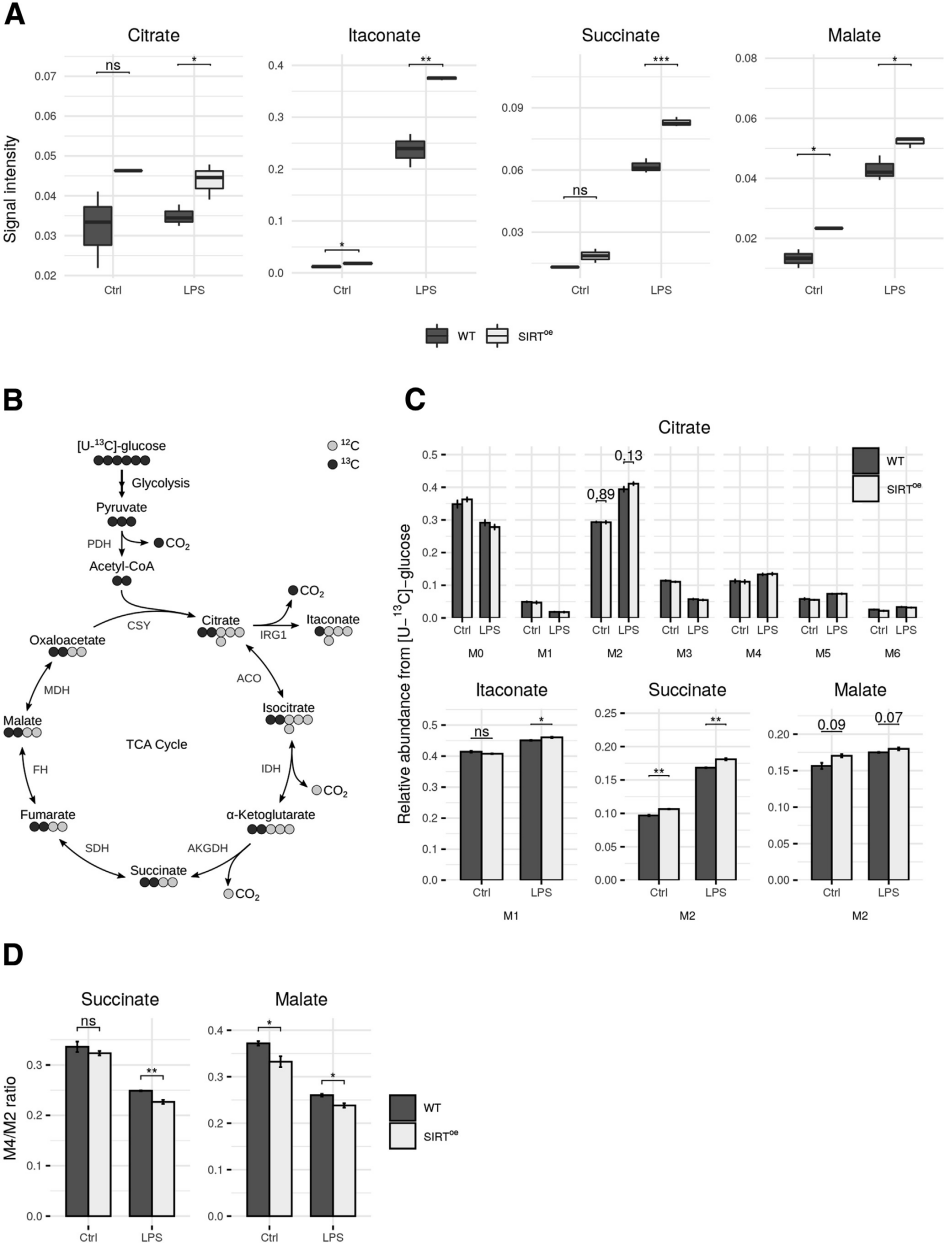
**Metabolome analysis of WT and SIRT**^**oe**^**BMDMs.** (*A*) Analysis of intracellular metabolite abundances. Signal intensities (peak area) were normalized to an internal standard D6-glutaric acid. (*B*) Scheme of atom transitions in the TCA cycle using a [U-^13^C]-glucose tracer. ^12^C-carbons are illustrated in light gray and ^13^C-carbons in dark gray. (*C*) MID of citrate as well as the relative abundances of M1-itaconate and M2-succinate and M2-malate. (*D*) Ratio of M4/M2-isotopologues of succinate and malate, indicating TCA cycling rate. Error bars indicate SE. Statistical significance was determined via Student’s *t* test (∗*P <* .05; ∗∗*P <* .01; ∗∗∗*P <* .001; control [Ctrl] n = 3, SIRT^oe^ n = 2). ACO, aconitase; AKGDH, α-ketoglutarate dehydrogenase; CSY, citrate synthase; FH, fumarate hydratase; ns, not significant; PDH, pyruvate dehydrogenase; IDH, isocitrate dehydrogenase; IRG1, aconitate decarboxylase; MDH, malate dehydrogenase; SDH, succinate dehydrogenase.

To elucidate the effect of SIRT^oe^ in LPS-activated macrophages, we first measured intracellular levels of TCA cycle–related metabolites. As expected, we found increased levels of citrate, itaconate, succinate, and malate after LPS activation (Figure 8*A*). Interestingly, the levels of itaconate and malate were already increased in resting macrophages overexpressing SIRT1 (Figure 8*A*). This effect was more pronounced in LPS macrophages; especially the significantly increased levels of itaconate and succinate indicating a further activation of SIRT^oe^ macrophages (Figure 8*A*).

To further investigate TCA cycle metabolism, we performed stable isotope labeling experiments and incubated SIRT^oe^ and WT BMDMs in the presence of a [U-^13^C]-glucose tracer. These experiments grant insight into intracellular glucose derived fluxes, as the labelled glucose is metabolized within the cells and the carbon isotopes are incorporated in downstream metabolites, resulting in specific enrichment patterns (Figure 8*B* for atom transitions).^46^^,^^47^

LPS stimulation resulted in an increased flux of glycolytic carbon through PDH, displayed by an increased fraction of M2 citrate, M1 itaconate, M2 succinate, and M2 malate isotopologues. This flux was even further increased in SIRT^oe^ BMDMs as compared with WT cells. The increased fraction of M1 itaconate isotopologues indicated an even higher synthesis rate of itaconate in SIRT^oe^ BMDMs, which agrees with the increased itaconate concentrations (Figure 8*C*).

In addition, we analyzed the TCA cycling flux by calculating the ratio of M4 to M2 isotopologues of TCA metabolites. We observed a significant reduction of TCA cycle activity under LPS stimulation, which was further attenuated in SIRT^oe^ BMDMs, although the glycolytic flux into the TCA was increased (Figure 8*D*). One reason for this reduction could be the depletion of NAD+ pools by SIRT1 activity. All the previously described findings indicate the rewiring of the TCA cycle, which is specific for LPS activation.^8^ As the effects are consistently stronger in SIRT^oe^ BMDMs, SIRT1 therefore seems to increase the proinflammatory response in macrophages.

### Overexpression of SIRT1 in Myeloid Cells Actively Contributes to Liver Injury and Fibrosis During Cholestasis

During chronic liver disease, infiltrating, proinflammatory macrophages dominate the liver macrophage pool and actively contribute to disease progression and fibrosis.^4^, ^5^, ^6^ Thus, while the inhibition of infiltrating macrophages attenuates the fibrotic response,^48^ the transfer of anti-inflammatory macrophages effectively reduces liver fibrosis in mice.^49^

**Figure 9:**
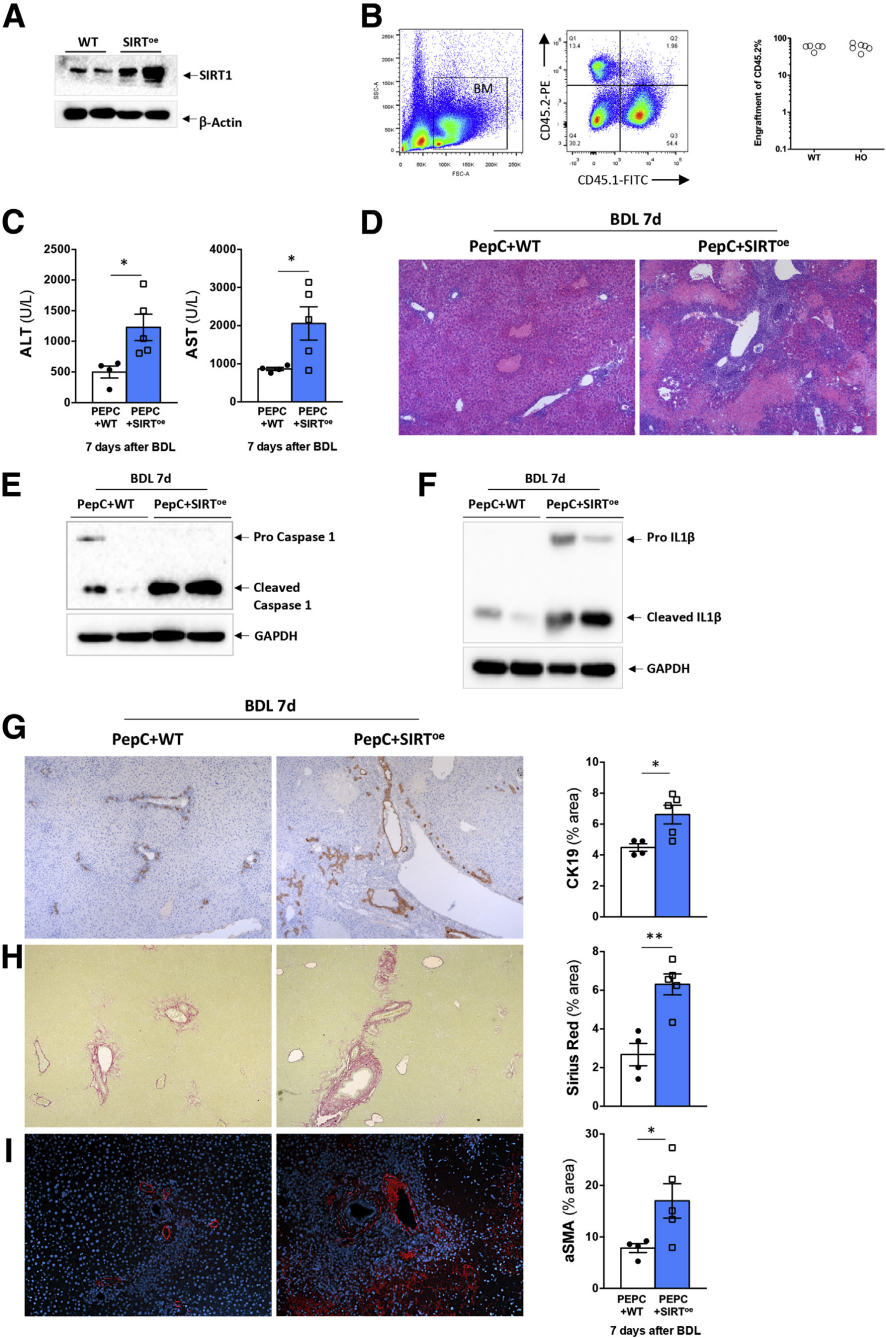
**Adoptive transfer of SIRT**^**oe**^**bone marrow cells contributes to cholestatic disease progression after BDL.** (*A*) Western blot analysis showing that bone marrow–derived cells from SIRT^oe^ have increased SIRT1 expression when compared with cells obtained from WT mice. (*B*) Fluorescence-activated cell sorting analysis of bone marrow cells isolated from WT (CD45.2) showing cell engraftment in recipient PEPC mice (CD45.1). (*C*) Serum levels of liver injury markers and (*D*) hematoxylin and eosin staining of liver sections from PEPC-Boy mice receiving bone marrow cells from WT or SIRT^oe^ mice analyzed at 7 days after BDL show increased liver injury in SIRT^oe^/PEPC mice. Western blot analysis of whole liver lysates showing increased (*E*) caspase-1 cleavage and (*F*) IL-1β cleaved protein expression in SIRT^oe^/PEPC mice 7 days after BDL. (*G*) Immunohistochemistry using an anti-CK19 antibody in paraffin-embedded liver sections showing enhanced ductular reaction in SIRT^oe^/PEPC mice compared with WT/PEPC mice. (*H*) Liver fibrosis was assessed by Sirius Red staining and (*I*) α-smooth muscle actin immunofluorescence on liver sections from transplanted mice 7 days after BDL. Images at (*D*) ×4 and (*G–I*) ×10 are representative of n = 4–5 animals/treatment group. Values are mean ± SEM. ∗*P <* .05, ∗∗*P <* .01, ∗∗∗ *P <* .001 [WT/PEPC vs SIRToe/PEPC]).

We found that macrophages overexpressing SIRT1 were hyperactivated, which could contribute to the exacerbated liver parenchymal injury observed in cholestatic SIRT^oe^ mice.^27^ To determine the impact of SIRT1 overexpression in myeloid cells (Figure 9*A*), and not hepatocytes or other noninflammatory cells, during the liver response to cholestatic injury in vivo, we adoptively transferred LK (lineage-negative, cKIT-positive) cells from WT or SIRT^oe^ mice (expressing CD45.2) into PEPC-Boy–recipient mice (expressing CD45.1, herein PEPC) (Figure 9*B*). The differential expression of CD45 in the donor vs recipient mice allowed us to confirm the engraftment of donor cells in the PEPC recipient mice (Figure 9*B*). Four weeks after engraftment, we performed BDL in both PEPC+WT and PEPC+SIRT^oe^ mice and analyzed the liver parenchyma after 7 days. Our results showed that PEPC+SIRT^oe^ mice had increased liver injury (Figure 9*C*), with livers showing wide areas of necrosis (Figure 9*D*). This damaging phenotype associated with higher activation of the inflammasome as evidenced by increased cleaved caspase-1 (Figure 9*E*) and cleaved IL-1β (Figure 9*F*) compared with PEPC+WT mice after BDL. PEPC mice receiving SIRT^oe^ cells showed also increased ductular reaction evidenced by CK19 immunostaining (Figure 9*G*). Ultimately, we found increased fibrosis in PEPC+SIRT^oe^ mice compared with mice receiving PEPC+WT mice at 7 days after BDL, as evidenced by Sirius Red staining (Figure 9*H*) and α-smooth muscle actin immunohistochemistry on liver sections and further quantification (Figure 9*I*).

Overall, our results show that SIRT1 overexpression in myeloid cells contributes to cholestatic disease progression by aggravating liver injury and fibrosis.

## Discussion

In this study, we demonstrate that SIRT1 regulates liver inflammation by controlling the activation of macrophages in response to endotoxin and during cholestatic liver disease. Mechanistically, we show that the overexpression of SIRT1 associates with mTOR activation, metabolic rewiring, the activation of the inflammasome, and attenuation of autophagy in macrophages. Ultimately, we demonstrate that the overexpression of SIRT1 in myeloid cells contributes to cholestatic disease progression, promoting liver damage and fibrosis.

Chronic liver disease progression is driven by hepatocellular cell death, triggering inflammation, fibrosis, and end-stage liver disease.^50^ In addition, liver disease associates with increased intestinal permeability that allows the translocation of bacteria (products) into the liver, aggravating inflammation and disease progression.^1^, ^2^, ^3^

Our previous and current results point to a dual mechanism by which SIRT1 overexpression contributes to cholestasis disease progression: (1) increased BA-mediated hepatocellular cell death,^27^ coupled with (2) increased inflammation mediated by the hyperactivation of macrophages overall aggravating cellular damage. The increased intestinal permeability observed in SIRT^oe^ mice could exacerbate macrophage activation and consequently hepatocellular liver damage. Our results in mice receiving SIRT^oe^ myeloid cells support the direct detrimental impact of these intrinsically hyperactivated proinflammatory macrophages in contributing to liver injury and cholestatic disease progression in vivo.

Our results pointing to a proinflammatory role of SIRT1 overexpression are in agreement with previous work in liver cancer in which SIRT1 promoted proinflammatory cytokine expression in macrophages.^51^ As well, the inhibition of sirtuins reduced the production of proinflammatory cytokines in human macrophages from rheumatoid arthritis patients^52^ and in immortalized macrophages in a NF-κB–dependent manner.^53^ Interestingly, these observations are in contrast to the anti-inflammatory effects reported by Yeung et al,^31^ in which the activation of SIRT1 by resveratrol inhibited NF-κB activation in vitro*.* Similarly, Pfluger et al^28^ showed that SIRT^oe^ mice had reduced inflammation when fed with a high-fat diet.

To gain mechanistic insights into the proinflammatory function of SIRT1, we observed in vivo (Figures 1 and 2), we performed in vitro studies on BMDMs in which we found that the overexpression of SIRT1 associated with a delayed p65 activation after LPS, rather than a complete inhibition, as described by Yeung et al.^31^ The different cell type used (non-small cancer cells) and the use of resveratrol, which can activate alternative pathways independently of SIRT1,^54^ could explain the discrepancy with our results.

Macrophages sense dying cells and bacteria via PRRs (eg, TLR) that activate the inflammasome leading to proinflammatory cytokine production.^12^ The regulation of the inflammasome and the production of IL-1β we observed in vivo and in vitro could result from the activation of different pathways, including the stabilization of Nlrp3 that can occur independently of NF-κB–mediated de novo protein synthesis.^55^ The activation of the inflammasome and IL-1β production can be also regulated by mTOR,^8^ a key mediator of the activation of macrophages.^8^^,^^16^^,^^20^^,^^56^ Accordingly, our results showed increased activation of the mTORC1 pathway in SIRT1^oe^ BMDMs in response to LPS that was supported by elevated pS6 and pULK^757^ expression. Moreover, our results showed that SIRT1-overexpressing macrophages were more dependent on mTOR to promote IL-1β production than WT cells in response to LPS. These results are interesting as the activation of SIRT1 with resveratrol has shown to inhibit the mTOR pathway,^57^ while others have described the synergy of SIRT1/mTORC1 activation in regulating cell response to stress.^58^ Likewise, the inhibition of SIRT1 related to suppressed mTOR activation and resulted in reduced inflammation and damage in the lung after infection.^59^ Notably, SIRT1 is necessary to activate mTOR downstream signaling via the deacetylation of S6K1, allowing its phosphorylation and activation of its kinase activity that phosphorylates ribosomal protein S6.^60^ This positive crosstalk was also described in human fibroblasts, in which overexpression of SIRT1 correlated with increased S6K1 phosphorylation.^61^ Most prominently, the coordinated activation of SIRT1 and mTORC1 promotes intestinal stem cell expansion in response to calorie restriction, in which deacetylation of S6K1 by SIRT1 enhances mTORC1 activation.^58^ Overall, our results support that SIRT1 and mTORC1 can cooperate in cell stress conditions in which cells require extensive energy like inflammation.

mTOR also regulates macrophage activation by inhibiting autophagy^20^ via mechanisms involving the inhibition of ULK1 by phosphorylation of serine 757.^11^ Autophagy is an essential mechanism to preserve cell metabolic and energy homeostasis as well as to control the host response to pathogens.^11^ Thus, autophagy controls the activation of the inflammasome and IL-1β production by mediating the degradation of pro-IL-1β.^10^ Likewise, impaired autophagy after loss of ATG16L1 enhanced the inflammasome activity and IL-1β production in macrophages after LPS^40^ and during Chron’s disease.^62^

The attenuation of autophagy we observe when SIRT1 is overexpressed could result from mTORC1-mediated negative regulation of autophagy via increased and sustained phosphorylation of ULK^757^, as we observed in LPS-treated SIRT^oe^ BMDMs. Alternatively, SIRT1 overexpression could regulate autophagy directly by contributing to the accumulation of p62, a negative regulator of autophagy.^41^ Indeed, our finding is in accordance with a recent study showing that the overexpression of SIRT1 in liver tumoral cells contributes to the accumulation of p62 and activation of mTOR, thus inhibiting autophagy and contributing to tumor progression and poor prognosis in patients.^63^ In turn, the stabilization of p62 in the context of SIRT1 overexpression could contribute to increase mTORC1 signaling in SIRT^oe^ BMDMs because p62 is essential to mediate the activation of the mTORC1 pathway via S6K1 activation.^64^

During the last decade, there have been remarkable advances in our understanding of the influence of metabolism in controlling immune cell activation, so-called immunometabolism. It is now known that during activation macrophages undergo a metabolic rewiring analogous to the Warburg effect occurring in cancer cells,^65^ in which aerobic glycolysis supports macrophage effector function.^7^ In addition to glycolysis, the activation of macrophages associates with the rewiring of the TCA cycle that leads to the accumulation of intermediates citrate, succinate, and fumarate,^7^ all with immunoregulatory functions. Citrate is key to produce itaconate, a potent antimicrobial^44^^,^^66^ by the downregulation of isocitrate dehydrogenase.^44^^,^^46^ Itaconate also supports inflammation by inhibiting succinate dehydrogenase and thus contributing to accumulation of succinate and sustained glycolytic reprograming.^43^ Succinate promotes glycolysis and proinflammatory cytokine production (IL-1β) in macrophages,^9^ providing an additional layer of interaction between cell metabolism and inflammation. Our results in BMDMs confirmed that the overexpression of SIRT1, and concomitant activation of mTORC1, promotes the metabolic rewiring of macrophages, characterized by increased use of glucose carbons in a broken TCA cycle and accumulation of immunomodulatory metabolites including citrate, succinate and malate. These findings, along with our observations in vivo, in which the adoptive transfer of SIRT1-overexpressing myeloid cells aggravates liver injury and fibrosis, support future studies in which the modulation of SIRT1 could be used as a strategy to rewiring macrophage metabolism and regulate inflammation. This strategy could as well reduce the inflammasome activation, which others and we have shown to contribute to liver injury and fibrosis during cholestatic disease.^1^^,^^2^^,^^14^^,^^15^^,^^33^ In line with this, previous work showed the efficacy of the adoptive transfer of anti-inflammatory macrophage to effectively reduce liver fibrosis in mice,^49^ and more recently, macrophage therapy has been established as clinically safe supporting the use of these promising approaches to treat chronic liver disease.^67^

Overall, our results provide novel mechanistic insights into the role of SIRT1 in regulating liver inflammation and warrant future research to define the potential of metabolic regulation of macrophages, via modulating SIRT1, as a therapeutic approach to treat cholestatic liver disease.

## Material And Methods

### Experimental Procedures in Animals

All experimental procedures were performed in 8- to 12-week-old male mice at the Disease Modelling Unit (University of East Anglia, Norwich, United Kingdom). All experiments were approved by the Animal Welfare and Ethical Review Body (University of East Anglia). All procedures were carried out following the guidelines of the National Academy of Sciences (National Institutes of Health, publication 86-23, revised 1985) and were performed within the provisions of the Animals (Scientific Procedures) Act 1986 and the LASA Guiding Principles for Preparing for Undertaking Aseptic Surgery (2010) under UK Home Office approval (70/8929).

SIRT^oe^ mice were generated on a C57/B6J background as previously described.^68^ Transplantation of isolated WT or SIRT^oe^ bone marrow cells into recipient PEPC-Boy mice was performed as previously described.^69^

Cholestasis was induced by BDL as described previously.^27^ Septic liver injury was induced by intraperitoneal injection of LPS (*Escherichia coli* E055:B5; Sigma-Aldrich, St. Louis, MO) at a dose of 20 mg/kg of body weight for up to 14 hours or by intraperitoneal administration of 35 mg/kg LPS and 700 mg/kg GalN (LPS/GalN) for up to 6 hours.

### Determination of Liver Damage

ALT and AST were determined in mouse serum using the Randox Daytona analyzer (Randox, Kearneysville, WV) following the manufacturer’s instructions, as previously described.^27^

### Isolation of Primary Hepatocytes

Primary hepatocytes were isolated as previously described.^27^ Primary cells were exposed to 100 ng/mL of LPS for 24 hours, after which apoptotic cell death was determined by caspase-3 activity assay, as previously described.^27^

### BMDM Differentiation and Culture

BMDMs were differentiated from bone marrow cells isolated from WT and SIRT^oe^ mice. The femur and tibia were flushed with RPMI media, and bone marrow cells were pelleted and plated with RPMI medium containing 10% fetal bovine serum and 30% L929 conditioned media and differentiated for 7 days. Fresh media was added at day 3 after plating. A total of 1 × 10^6^ adherent cells were then plated for experiments in 6-well plates.

BMDMs from WT and SIRT^oe^ mice were cultured in the presence of 100 ng/mL LPS for up to 24 hours. For determination of autophagy, all cells were pretreated with 20 mM NH_4_Cl and 100 μM leupeptin to inhibit lysosomal proteolysis, 2 hours before LPS treatment.

### Histology, Immunohistochemistry, and Immunofluorescence

Liver tissues were fixed in 10% neutral buffered formalin (Sigma-Aldrich). Liver tissues were embedded in paraffin and subsequent tissue blocks were sectioned, dewaxed, and hydrated. For pathological analysis, liver sections were stained with hematoxylin and eosin as described previously.^1^^,^^27^ Immunohistochemistry was carried out using an anti-CK19 antibody (Developmental Studies Hybridoma Bank, University of Iowa, Iowa City, IA), as described previously.^1^^,^^27^ Also, anti-CD11b (Abcam, Cambridge, United Kingdom) and anti-F4/80 (Bio-Rad ABD Serotec Limited, Oxford, United Kingdom) were used, as previously described.^70^ Quantification was carried out using Fiji software (v1.53f51) and is shown as the percentage of stained area relative to the total area per field. A total of 5–10 fields per sample were imaged and analyzed.

Fibrosis was determined using Sirius Red staining and IF using a Cy3-conjugated α-smooth muscle actin antibody (Sigma-Aldrich). For IF, nuclei were counterstained with DAPI. The quantification of collagen fibers was performed using Fiji software and is represented as the percentage of stained area relative to total area per field. A total of 5–10 fields per sample were imaged and analyzed.

IF imaging of liver sections was performed using anti-Nlrp3 (Santa Cruz Biotechnology, Dallas, TX), anti-CD11b (Abcam) and anti-Ly6C (Miltenyi Biotec, Bergisch Gladbach, Germany) primary antibodies followed by secondary Cy-3-anti-mouse (for Nlrp3) and FITC-anti-rabbit and FITC-anti-rat for CD11b and Ly6C respectively. Slides were counterstained and mounted with Vectashield Antifade mounting medium with DAPI (Vector Labs, Burlingame, CA).

For immunofluorescence imaging of BMDMs, cells were grown on glass coverslips and were fixed with ice cold 30% acetone 70% methanol for 15 minutes at 4°C. Cells were washed and blocked with 10% goat serum, 0.01% Triton X-100, and 1% bovine serum albumin (BSA) in phosphate-buffered saline (PBS). Cells were incubated overnight with anti-p65 (Santa Cruz Biotechnology) or LC3 (Cell Signaling Technology, Waltham, MA) in 1% BSA in PBS. Cells were washed and incubated for 1 hour with goat anti-rabbit Alexa Fluor 568 secondary (Thermo Fisher Scientific, Waltham, MA) in 1% BSA in PBS. Cells were washed and mounted using Vectashield Antifade mounting medium with DAPI (Vector Labs).

### Metabolic Determinations

Isolated and differentiated WT and SIRT^oe^ BMDM cell cultures were washed with 1XDPBS, followed by the addition of SILAC RPMI 1640 media (Gibco, Waltham, MA) supplemented with 11 mM of the 13C6 glucose tracer (Sigma-Aldrich) and 2 mM of 12C5 glutamine (Carl Roth, Karlsruhe, Germany) along with arginine and lysine, without fetal bovine serum. Cells were starved for 4 hours, following the addition of 100 ng/mL of LPS for 3 and 6 hours.

After 3- and 6-hour LPS incubation, plates were washed once with 2 mL of 0.9% NaCl (Sigma-Aldrich). Intracellular metabolites were extracted by adding 400 μL of methanol (–20°C) and 400 μL ddH_2_O (4°C) containing 1 μg/mL D6-glutaric acid (internal standard). Cells were scrapped and transferred to a tube containing 400 μL chloroform (–20°C) and mixed for 20 minutes at 1400 rpm and 4°C (Eppendorf ThermoMixer C; Eppendorf, Hamburg, Germany). Polar, aqueous, and nonpolar phases were separated by centrifugation (5 minutes, 21,000 *g*, 4°C), and 300 μL of the polar phase was transferred to GC glass vial. Vials were vacuum-dried at 4°C, capped and stored at –80°C for further analysis.

Derivatization of samples was performed with an Axel Semrau Chronect Robotic Pal RTC (Axel Semrau, Sprockhövel, Germany) directly before gas chromatography mass spectrometry measurement. A total of 15 μL of 2 % methoxyamine hydrochloride in pyridine was added to the samples following agitation for 60 minutes at 40°C. Afterward, an equal volume of N-methyl-N-(trimethylsilyl)trifluoroacetamide was added and shaking continued for 30 minutes at the same temperature. A total of 1 μL of each derivatized sample was injected in splitless mode into an SSL injector heated to 270°C. The gas chromatographic separation was performed on an Agilent 7890B GC (Agilent, Santa Clara, CA) equipped with a 30 m ZB-35 + 5 m DuraGuard column (Phenomenex, Torrance, CA). Helium was used as carrier gas with a flow rate of 1 mL/min. Initially, the gas chromatography oven temperature was held at 80°C for 6 minutes. Afterward, the temperature was raised by 6°C/min until 300°C were reached, and was finally held for 10 minutes. Then, the temperature was increased to 325°C at 10°C/min and was held for additional 4 minutes, resulting in a total run time of 59.167 minutes for 1 sample. The gas chromatography system was coupled to an Agilent 5977B MSD. Electrical ionization of the metabolites was performed at 70 eV. The mass spectrometry ion source was constantly heated to 230°C and the quadrupole to 150°C. For the untargeted approach, full-scan mass spectra were acquired from 70 m/z to 800 m/z. For the labeling experiments, the connected detector was operating in selected ion monitoring. Gas chromatography mass spectrometry chromatograms were processed using the inhouse developed software MetaboliteDetector, v3.320200313.^71^ Mass isotopomer distributions were calculated according to the chemical formulas from Wegner et al.^72^

### RNA Isolation and Quantitative Real-Time Polymerase Chain Reaction

RNA was isolated from liver samples or cell cultures using QiAzol lysis Reagent (Qiagen, Hilden, Germany). First strand synthesis and reverse transcription was performed using M-MLV Reverse Transcriptase (Invitrogen, Waltham, MA). Quantitative real-time polymerase chain reaction was carried out using SYBR Green reagent (Life Technologies, Carlsbad, CA) using the ViiA7 real-time polymerase chain reaction detection system (Applied Biosystems, Waltham, MA). Gene expression was normalized to TBP1 and is represented in times vs control sample gene expression. Primer sequences can be provided upon request.

### Western Blot Analysis and Enzyme-Linked Immunosorbent Assay

Proteins were extracted from snap frozen livers or cultured BMDMs using RIPA buffer containing 50 mM Tris-HCL, 150 mM NaCl, 0.1% sodium dodecyl sulfate, 2 mM EDTA, 5% sodium deoxycholate, 1% Igepal 630, 1 mM PMSF, and protease inhibitor (Sigma-Aldrich).

Proteins were resolved using sodium dodecyl sulfate-polyacrylamide gels and transferred to nitrocellulose membranes (Whatman; Sigma-Aldrich). Membranes were probed with IL-1β, caspase-1, Nlrp3, TLR2, and TLR4 (all from Santa Cruz Biotechnology), as well as with PARP, pS6, p62, pULK^757^, and LC3 A/B (all from Cell Signaling Technology). β-actin (Sigma-Aldrich), GAPDH, or α-tubulin (Abcam) were used as loading controls. We used anti-rabbit IgG-HRP–linked or anti-mouse IgG-HRP linked secondary antibodies (Cell Signaling Technology).

Expression of IL-1β was determined in BMDM protein extracts while IL-10 and IL-4 were determined in whole liver protein extracts using R&D Systems DuoSet (R&D Systems, Minneapolis, MN) according to the manufacturer’s instructions.

### Flow Cytometry

Immune cells were isolated from mouse liver, as described previously.^1^^,^^27^ Immune cells were stained with CD45-APC-Cy7 (Becton Dickinson, Franklin Lakes, NJ), CD11b-PE (Becton Dickinson), F4/80-FITC (Miltenyi Biotec), and Ly6C-Pacific blue (MACS). Flow cytometry was carried out using BD LSR-Fortessa. Analysis was performed using FlowJo software (FlowJo 10.8.1, Ashland, OR).

## Statistical Analysis

Data are expressed as mean ± SEM. Statistical significance was determined using 2-way analysis of variance followed by Student’s *t* test or Student’s *t* test only, as appropriate, using GraphPad Prism 6 software (GraphPad Software, San Diego, CA).
